# Depression and Anxiety During the COVID-19 Pandemic: Epidemiology, Mechanism, and Treatment

**DOI:** 10.1007/s12264-022-00970-2

**Published:** 2022-11-21

**Authors:** Chunyan Zhu, Ting Zhang, Qianqian Li, Xingui Chen, Kai Wang

**Affiliations:** 1 School of Mental Health and Psychological Sciences, Anhui Medical University, Hefei, 230032 China; 2 Anhui Province Key Laboratory of Cognition and Neuropsychiatric Disorders, Hefei, 230032 China; 3 Collaborative Innovation Center of Neuropsychiatric Disorders and Mental Health, Hefei, 230032 China; 4 Department of Psychiatry, The First Affiliated Hospital of Anhui Medical University, Hefei, 230032 China; 5 Department of Neurology, The First Affiliated Hospital of Anhui Medical University, Hefei, 230032 China; 6 Institute of Artificial Intelligence, Hefei Comprehensive National Science Center, Hefei, 230032 China

**Keywords:** COVID-19, Anxiety, Depression, Epidemiology, Mechanism, Intervention

## Abstract

The Coronavirus Disease 2019 (COVID-19) pandemic has had an adverse impact on the physical and mental health of the public worldwide. In addition to illness in patients with COVID-19, isolated people and the general population have experienced mental health problems due to social distancing policies, mandatory lockdown, and other psychosocial factors, and the prevalence of depression and anxiety significantly increased during the pandemic. The purpose of this review is to elucidate the epidemiology, contributing factors, and pathogenesis of depression and anxiety. during the pandemic. These findings indicate that physicians and psychiatrists should pay more attention to and identify those with a high risk for mental problems, such as females, younger people, unmarried people, and those with a low educational level. In addition, researchers should focus on identifying the neural and neuroimmune mechanisms involved in depression and anxiety, and assess the intestinal microbiome to identify effective biomarkers. We also provide an overview of various intervention methods, including pharmacological treatment, psychological therapy, and physiotherapy, to provide a reference for different populations to guide the development of optimized intervention methods.

## Introduction

Since the emergence of Coronavirus Disease 2019 (COVID-19) in December 2019, the severe acute respiratory syndrome coronavirus-2 (SARS-CoV-2) has infected over 500 million people worldwide (World Health Organization. https://covid19.who.int/). Severe respiratory symptoms, high mortality rates, and rapid transmissibility have made COVID-19 a severe illness that has had a negative impact on both physical and mental health [1, 2]. During the pandemic, many people have experienced severe anxiety and fear of getting sick, which has led to a series of mental health symptoms, including lack of motivation, anhedonia, exhaustion, irritability, and sleep disturbance. Depression and anxiety during COVID-19 have been major causes of a global health-related burden and will have long-term economic and social consequences [2].

Recent literature from the COVID-19 Mental Disorders Collaborators indicates that the prevalence and burden of depression and anxiety has increased significantly during the pandemic [2]. They estimated the addition of 53.2 million new patients with major depressive disorder and 76.2 million cases of new anxiety disorders globally throughout 2020. Meanwhile, they found that daily SARS-CoV-2 infection rates and reduced mobility were associated with increased prevalence of depression and anxiety. As SARS-CoV-2 evolved, variable virus strains caused illness with differences in the severity of pneumonia, treatment approaches, vaccination, and control measures, and subsequent COVID-19-related mental health problems also varied. A new variant with rapid transmissibility, SARS-CoV-2 Omicron, resulted in a new worldwide outbreak, resulting in mental health problems that differed from those prevalent at the beginning of the pandemic [3].

**Fig. 1:**
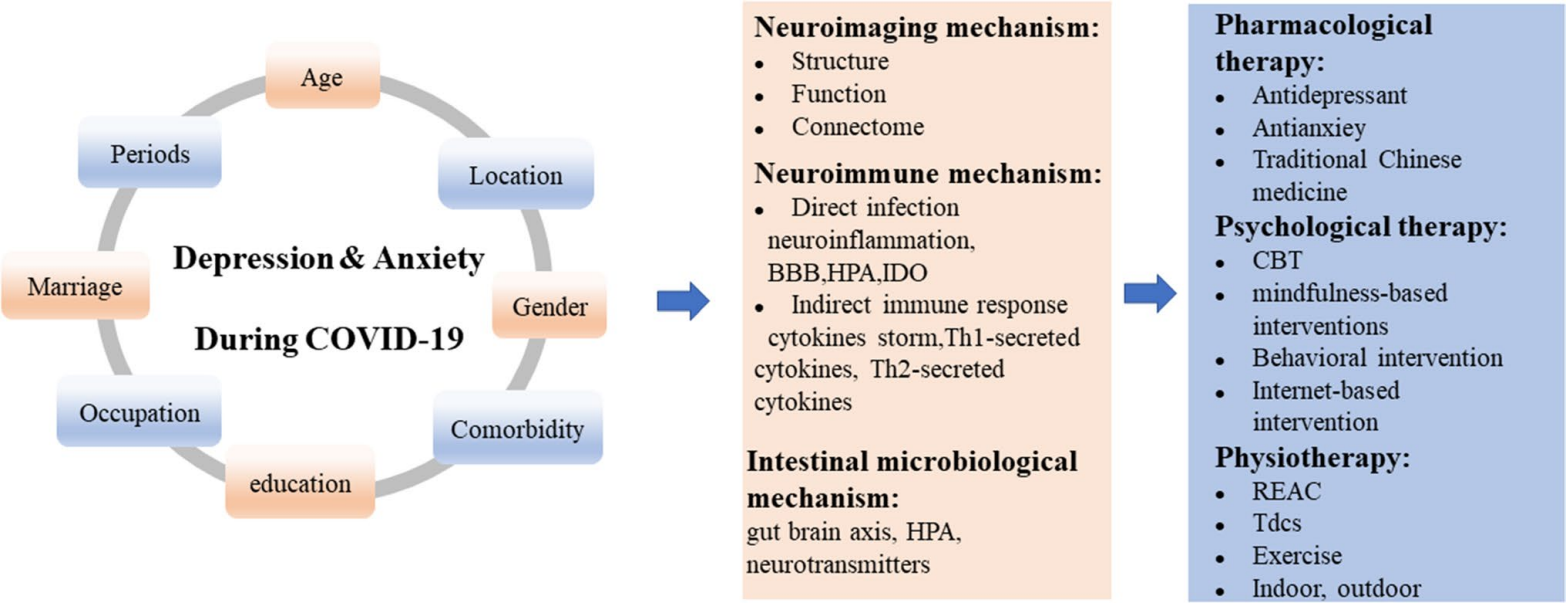
Depression and anxiety during COVID-19: epidemiology, mechanisms, and treatment. BBB, blood-brain-barrier; HPA, hypothalamic-pituitary-adrenal; IDO, indoleamine 2,3-dioxygenase; CBT, cognitive behavioral therapy; REAC, radio electric asymmetric conveyer; Tdcs, transcranial direct current stimulation

The purpose of this review is to elucidate the epidemiology, contributing factors, and pathogenesis of depression and anxiety during the pandemic and to discuss mechanisms and treatments. Recently, studies focusing on the etiology of COVID-19-related depression and anxiety suggested that changes in brain structure and inflammation may be the underlying mechanisms responsible for depression and anxiety, although the exact mechanism remains unclear. Probing the neural mechanism is imperative for the diagnosis and treatment of COVID-19-related depression and anxiety. We also summarize pharmacological treatments, psychological therapy, and physiotherapy to provide guidance for clinicians (Fig. 1).

## Epidemiology

Current literature reports the pooled prevalence of depression and anxiety is 45% and 47% respectively, which is higher than during the non-epidemic period [4]. Populations present some level of worry about COVID-19, regardless of infection status, and the level depends on various contributing factors. We have searched recent literature concerning the mental health problems during COVID-19 and found several risk factors. Consistent findings included age, gender, the periods of the pandemic, location, different populations, educational level, profession, marriage, and comorbidities. In this review, we summarized these contributing factors to provide a potential direction for future research on mental health problems.

### Age and Gender

Age and gender had a significant effect on the levels of risk for depression and anxiety during the pandemic. Consistent studies have shown that younger people have greater vulnerability to mental diseases [5–7]. Zhou *et al*. reported the prevalence of depressive and anxiety symptoms to be 43.7% and 37.4%, respectively, among Chinese high school students during the COVID-19 outbreak [5]. A longitudinal study indicated that adolescents exhibited more depression and anxiety during the two months following the implementation of government restrictions and online learning [8]. Ahmed *et al*. investigated these psychological problems during the pandemic among all ages and found that young people aged 21–40 years were more vulnerable in terms of their mental health conditions [6]. The anxiety risk of people older than 40 years was only 0.40 times higher than that of younger people [9]. The frequency of social media exposure (SME) might be responsible for this difference in anxiety risk [7]. In another study, older people (>50 years) were reported to be another group at risk for developing mental problems [10], possibly due to loneliness, lack of physical activity, and ageism [11, 12]. The inconsistency among results may be related to different races and evaluation methods in the different studies.

Gender is another demographic characteristic that has been reported to as a factor associated with mental health problems. A number of studies reported that being female increased vulnerability to depression and anxiety during the pandemic [13–18]. Wang *et al*. conducted a cross-sectional trial among the general population in China that included 600 respondents, and found that the risk of anxiety in females was three times higher than that in males [9]. The vulnerability of females may be due to the higher proportion of SME [7]. Many women worked in healthcare during the pandemic or took care of their families, and the COVID-19 pandemic and quarantine policies might have significantly affected their lifestyle and led to greater worries [19]. Especially, pregnant women were more affected [20], with a 37% incidence of depression and 57% incidence of anxiety. Pregnant women tended to fear that COVID-19 would threaten both their life and that of their baby, and they worried about obtaining necessary prenatal care and social isolation [21]. However, limited inconsistent findings suggested that male participants displayed a higher risk for depression and anxiety [22]. Many factors could explain differences between males and females, such as differences in participation in risky behaviors (e.g., going to crowded places or not wearing masks) during the pandemic and differences in the infection rate between males and females. In addition to those social factors, biological underpinnings also played a critical role in women’s susceptibility to depression and anxiety. For example, gonadal hormones contributed to the diathesis factor of emotional dysregulation being over-represented in females [23].

### Different Periods of the Pandemic and Different Populations

The prevalence of depression and anxiety have varied throughout the different periods of the pandemic. There was a spike in the number of cases over a short period of time between January 23 and March 10, 2020 in China, which can be called an outbreak. Wang *et al*. conducted a longitudinal study and found that the rates of depression and anxiety were 16.5% and 28.8% during the initial outbreak of COVID-19 [24]. Their subsequent evaluation showed no significant longitudinal changes in the rates of mental disorders compared to the initial evaluation. However, a large-scale online survey during the outbreak reported higher rates [22]. Another study reported rates of depression and anxiety of 27.9% and 31.6%, respectively, among the survey respondents, markedly higher than pre-pandemic levels [25]. The elevated prevalence may be due to fear of infection and home quarantine [22]. Since March 10, 2020, COVID-19 in China has been basically under control, with newly-confirmed cases in Hubei Province at zero, and newly-confirmed cases nationwide showing a consistent downward trend. This phase was considered to be a period of remission. During the remission period, there was a persistent increase in the level of depression and anxiety among Chinese college students, with a longitudinal survey reporting elevated rates relative to values during the onset of the outbreak (21.6% *vs* 26.3% for depression, 11.4% *vs* 14.7% for anxiety) [26]. Less physical exercise and social support were found to be associated with psychological symptoms [26]. Zaninotto *et al*. reported that mental health began to recover in July through October 2020, but the prevalence of depression and anxiety then increased further by the end of 2020 compared to June 2020 [27]. This trend might be associated with increased loneliness and decreased quality of life during the lockdown phases [14, 27]. These findings suggest that mental health problems during different stages of the pandemic could be attributed to different psychosocial factors, which need to be recognized as early as possible.

Besides, different populations varied in the prevalence of depression and anxiety, and this may be associated with the possibility of epidemic exposure. Mazza and colleagues reported the prevalence rates of depression and anxiety were 31% and 42% in COVID-19 survivors [28]. Furthermore, the prevalence of depression and anxiety was reported to be highest among patients with COVID-19 infection 55%[48%–62%] and 56%[39%–73%] [29]. They had higher levels of depression and anxiety (both *P* <0.001), compared to non-COVID controls [30]. Notably, non-COVID populations also displayed a significantly increased prevalence of depression and anxiety. A cross-sectional online study during self-quarantine showed that 50.9% of participants had traits of anxiety and 58.6% exhibited depression [31]. Results from multiple countries suggested that the pooled prevalence of depression was 21.7% (95% CI, 18.3%–25.2%), and of anxiety was 22.1% (95% CI, 18.2%–26.3%) in health-care workers [32], which was similar to that in general populations. Hence, it is necessary to pay different levels of attention to the mental health problems of the various populations.

### Education, Living Location, Profession, and Marital Status

In addition to demographic features, socioeconomic factors might affect the prevalence of depression and anxiety during the COVID-19 pandemic. Several studies reported that educational level was associated with the risk of mental disorders. Zhao *et al*. found that people with low levels of education (senior high school and below) showed more anxiety symptoms than those with higher levels (college and above) [33]. A study in Australia also suggested that a high educational level was protective against depression [34]. Furthermore, a cross-sectional investigation in China showed that the risk of depression for those with a master’s degree was only one-third that of those with a bachelor’s degree [9]. However, a cross-sectional study in Bangladesh found that individuals with a higher educational level (above college) had more anxiety symptoms [35]. Differences in ethnicity and self-rating scales may account for the inconsistent results.

Living location is another factor associated with depression and anxiety during COVID-19. Several studies showed that people living in rural areas had higher levels of anxiety [26, 35]. Rural people had a higher frequency of SME, which was correlated with the prevalence of mental health problems [26]. Lower levels of economic security, material security, and sanitary conditions also contributed to the problem in rural areas [35]. However, urban residents were also at risk of developing depression and anxiety symptoms [11, 36]. The majority of COVID-19 cases are occurring in urban areas [36]. Cities are more densely populated, and therefore more susceptible to novel coronavirus transmission. A study conducted in 204 countries estimated large increases in the prevalence of mental health conditions in Latin America, the Caribbean, North Africa, the Middle East, and South Asia [2]. Zheng *et al*. reported that the proportion of severe depression during the pandemic in Hubei Province was more than double that of other provinces in China during the outbreak [37]. Overall, the mental health problems were associated with the COVID-19 control strategies and social distancing policies in the province. These results indicate that agencies should strengthen supervision over social media news and guarantee the accuracy of reporting on the epidemic situation.

Moreover, recent studies have revealed an association of profession with susceptibility to depression and anxiety during COVID-19, especially in front-line healthcare workers, migrant workers, and workers in contact with the public [36, 38]. Zhang *et al*. found a higher prevalence of insomnia (38.4% *vs* 30.5%), anxiety (13.0% *vs* 8.5%), and depression (12.2% *vs* 9.5%; *P* <0.04) in medical health workers compared with non-medical health workers [36]. Another study by Liu and colleagues reported that medical staff who had direct contact treating infected patients experienced higher anxiety scores than those who had no direct contact [38]. For migrant workers, the dominating anxiety was from the suspension of productive activity, loss of income, and fear of the future. For workers in contact with the public, anxiety was a possible consequence of being exposed to infection every day. Notably, populations with a stable family income and living with the family reported fewer mental problems. Therefore, we ought to pay more attention to staff in these special occupations, make psychological measurements, and provide interventions.

The effect of marital status on mental health problems varied across studies. Several studies reported more anxiety, depression, and insomnia in married than in unmarried people [7, 16, 17, 35]. Fu *et al*. found that married people frequently worry more about their family’s health than about their own health [17]. However, Shi *et al*. found a higher risk of depression and anxiety in unmarried people [22]. Two studies reported that divorce or widowhood was an important predictor of the levels of depression and anxiety [33, 39], possibly as a consequence of greater loneliness and the lack of emotional support [33, 34]. Discrepancies among studies in terms of pandemic period, location, and measurement tools may account for the inconsistency of results.

### Comorbidity

Comorbidity of chronic diseases has been another risk factor for mental health problems during the COVID-19 pandemic [24, 34, 36, 40]. A multinational and multicenter study conducted by Chew *et al*. reported that healthcare workers with comorbid chronic diseases, such as hypertension, hyperlipidemia, and diabetes mellitus, had greater susceptibility to psychological problems than those without comorbidities [41]. Lotzin *et al*. found that comorbidity of psychiatric diseases increased people’s vulnerability to depressive and anxiety symptoms during the pandemic [42]. The increased difficulty in accessing medical care during the pandemic is an important reason for the increase in depression and anxiety associated with chronic illness [42, 43]. For people with comorbid chronic diseases who are susceptible to mental health problems, regular psychological assessments should be conducted at the same time as the underlying diseases are being treated.

## Mechanism

Considering the high prevalence of depression and anxiety during the COVID-19 pandemic, researchers have focused on identifying the mechanism responsible for these illnesses and on better understanding, recognition, and treatment. As a non-invasive technology, functional magnetic resonance imaging has been applied to explore the neural mechanism that underlies depression and anxiety. Pre-pandemic brain function, structure, and connectome have been reported to be predictive factors for depression and anxiety during the pandemic. Recent studies also showed the imperative roles of the direct and indirect immune response on the neuroimmune substrates involved in depression and anxiety during COVID-19. In addition, several studies reported an association between intestinal microbiota and mood disorders.

### Neural Mechanism

Recently, researchers have probed the neural mechanisms involved in depression and anxiety during the COVID-19 pandemic. The major findings focused on brain structural morphology, functional activity, and neural networks. Abnormal structure in the limbic region has been reported in patients with depression and anxiety. Holt-Gosselin *et al*. found that reduced thickness in the insula before the pandemic predicted more severe anxious arousal symptoms during COVID-19. Anhedonia, as predicted by self-distraction, interacts with amygdala volume [44]. In a longitudinal investigation, Salomon *et al*. found increased volume in the bilateral amygdala, putamen, and anterior temporal cortices following the COVID-19 outbreak and lockdown, but the volumes decreased as time elapsed after lockdown [45]. This finding suggests that the intense experience associated with the pandemic induced transient volumetric changes in brain regions commonly associated with stress and anxiety. In another study, Jamieson *et al*. found that structural integrity of the posterior limb of the internal capsule pre-pandemic was associated with worry and rumination during COVID-19 [46]. These results suggest that early brain structural changes were predisposed to trigger anxiety or depressive symptoms during the onset of COVID-19.

Based on a functional neuroimaging study, Khorrami *et al*. suggested that greater anterior insular activation in response to unpredictable threat and greater self-reported intolerance of uncertainty are independent predictors of increased pandemic-related negative affect [47]. Du *et al*. reported that altered amplitude of low-frequency fluctuation in regions related to emotion and sleep regulation occurred in COVID-19 survivors [48], and Zhang *et al*. reported that decreased functional connectivity of amygdala subregions predicted vulnerability to depression following the pandemic [49]. A long-term longitudinal study using 18F-FDG-PET/CT reported lasting prefrontal, insular, and subcortical metabolism in COVID-19 patients with anxio-depressive symptoms [50]. However, functional neuroimaging investigations conducted during COVID-19 are limited, and most studies were retrospective. Future observational and prospective investigations are needed.

The functional connectome is another potential mechanism that might underlie pandemic-related depression and anxiety disorders. He *et al*. reported that the pre-pandemic functional connectome could predict pandemic-related anxiety [51]. They proposed that weaker connectivity between the executive control network and the salience network might account for increased signs of pandemic-related anxiety. Those with lower top-down executive control [52] failed to inhibit or regulate somatic and autonomic bodily states [53, 54] and the hyperactivity caused by detecting and filtering salient stressful events [55]. He *et al*. also found that another neural circuit involving the insula, thalamus, hippocampus, and parahippocampal gyrus and the sensorimotor cortex was associated with higher pandemic-related anxiety. This evidence emphasizes that the pandemic-related anxiety may result from distributed neural circuits.

### Neuroimmune Mechanism

Current literature suggests that the interaction between neurocircuits and neuroinflammation drives the development of depression. Therefore, the neuroimmune response may have played a critical role in depression during the pandemic. Ellul *et al*. reported that direct infection of the central nervous system can induce psychological symptoms due to the potential neurotropism of coronaviruses [56]. Dantzer *et al*. reported that neuroinflammation, blood-brain-barrier disruption, peripheral immune cell invasion into the central nervous system, neurotransmission impairment, hypothalamic-pituitary-adrenal (HPA) axis dysfunction, microglial activation, and indoleamine 2,3-dioxygenase induction were involved in the neuroimmune mechanism of depression and anxiety development during the pandemic [57–60].

In addition to direct viral infection, an indirect immune response to viral infection may have played an equivalent role in the development of mental symptoms during the pandemic. For example, Pedersen reported that the virus can trigger a cytokine storm that induces a series of immune responses [57, 61]. The production of cytokines, chemokines, and other inflammatory mediators increases both locally and systemically [62]. COVID-19 patients showed elevated levels of interleukin (IL)-1β, IL-6, interferon (IFN)-γ, CXCL10, and CCL2, suggesting activation of T-helper-1 cell function. Several studies indicated that the amount of IL-6 and IL-1β may be related to the risk of developing post-COVID depression. Moreover, patients with COVID-19 displayed higher levels of T-helper-2 cell-secreted cytokines (such as IL-4 and IL-10) [63, 64]. Higher concentrations of these cytokines may predict a worse clinical course [65]. Notably, the dysregulation of IL-1β, IL-6, IL-10, IFN-γ, tumor necrosis factor alpha, and transforming growth factor-β is associated with psychiatric diseases [66–70]. Nevertheless, the evidence described above came mostly from cross-sectional investigations, and longitudinal changes need to be evaluated. In the future, cohort studies are needed to determine the changes in neuroimmune factors related to post-COVID depression and anxiety symptoms in order to identify biomarkers.

### Intestinal Microbiological Mechanism

The association between intestinal microbiota and mood disorders is currently a hot research topic. Numerous studies have shown that depression and anxiety are associated with an imbalance of intestinal flora, which leads to abnormality in the gut-brain axis [71, 72]. Ghannoum *et al*. reported that altered intestinal flora in the multiple dimensions of the gut-brain axis had a negative impact, including excessive activation of the HPA axis (cortisol), neural circuits, and the level of neurotransmitters such as dopamine and serotonin, as well as excessive production of pro-inflammatory cytokines in the immune system (e.g., IL-6) and the destruction of the intestinal barrier [71]. Eventually these changes contributed to depression and anxiety. Nakov *et al*. reported an increased prevalence of gastrointestinal symptoms during the COVID-19 lockdown [73]. They found that the occurrence of these symptoms was associated with dysfunction of the gut-brain axis, thereby causing changes in the neuroimmune and endocrine systems and promoting the development of depression and anxiety. In an animal study, Tian *et al*. showed that *Lactococcus* CCFM6432 effectively reduced stress-induced anxiety behavior by alleviating overactivation of the HPA axis and improving the composition of intestinal flora [74]. These studies indicate that COVID-19 infection can cause depression and anxiety symptoms by changing the intestinal microbiome. These findings provide direction for developing interventions to treat COVID-19-related depression and anxiety.

## Treatment

Depression and anxiety during the COVID-19 pandemic have had a profound impact on people's lives, and in response, researchers placed a high priority on interventions. Pharmacological therapy, such as antidepressant and antianxiety drugs, play a critical role in the intervention for depression and anxiety. In addition, psychological interventions such as cognitive behavioral therapy (CBT), mindfulness-based interventions, progressive muscle relaxation training, and internet-based interventions played an important role in alleviating psychological problems during COVID-19 [75–78]. Another effective treatment was physical therapy, as neuromodulation and exercise have been shown to alleviate depressive and anxiety symptoms [79].

### Pharmacological Therapy

Selective serotonin reuptake inhibitors and selective serotonin and norepinephrine reuptake inhibitors are the most commonly used antidepressants, but to date no longitudinal clinical efficacy studies of their use during the pandemic have been published. Although ketamine is rarely used clinically, Rosenblat *et al*. reported that it could relieve depressive symptoms, suicidal ideation, and anxiety symptoms in patients with depression both pre- and post-pandemic [80].

Anxiety disorders are usually treated with antidepressants and benzodiazepines, and these were the main drugs used to treat anxiety during COVID-19. During the pandemic, it was particularly crucial to control acute anxiety attacks in patients infected with COVID-19. Khawam *et al*. found that administering alprazolam reduced the risk of respiratory depression and acute respiratory failure in COVID-19 patients with acute anxiety [81]. Besides, olanzapine, quetiapine, or haloperidol were effective in treating anxiety disorder [81]. Based on traditional Chinese medicine theory, Ma *et al*. found that Suanzaoren Decoction, Huanglian Ejiao Decoction, and Zhizi Chi Decoction reduced anxiety symptoms [82].

Although the use of classic antidepressant and antianxiety drugs during the pandemic were less reported, empirical treatment has evidenced their confirmed efficacy in depression and anxiety. For the newer drugs, we need more information that is based on the actual situation of patients and the monitoring of adverse effects.

### Psychological Interventions

The COVID-19 pandemic has placed tremendous mental health stress on people [83]. Psychological interventions are often regarded as the fundamental treatment for mental disorders, and several studies focused on such interventions during COVID-19, including CBT, mindfulness-based interventions, behavioral interventions, and internet-based interventions.

Recent studies have shown that CBT was effective at relieving depressive and anxiety symptoms in patients with COVID-19 [75, 84, 85]. Li *et al*. found a positive effect of face-to-face CBT on improving the mental health of patients with COVID-19 [75]. Liu *et al*. reported that computerized CBT and face-to-face interventions had similar efficacy [84]. Due to lower net societal costs and reduced risk of exposure [86], internet-delivered CBT can be a superior psychological therapy during the pandemic.

Several investigations have reported the efficacy of mindfulness-based interventions on mental health problems [76, 87–90]. Maria Antònia *et al*. reported that the PsyCovidApp group showed significant improvements in insomnia, anxiety, and stress [87]. Malboeuf-Hurtubise reported that mindfulness-based intervention enhanced the basic psychological need satisfaction for elementary school students [90].

As a behavioral intervention, progressive muscle relaxation training was shown to improve sleep quality and reduce depression and anxiety in patients with COVID-19 [77, 91]. Similarly, depressive and anxiety symptoms were significantly reduced *via* 10-day psychological support and breathing exercises [92]. Therefore, we recommend that this behavioral intervention be introduced to the general hospital setting during the pandemic.

Current evidence suggests that internet-based integrated intervention can decrease the levels of anxiety, depression, and perceived stress and increase resilience [78, 93]. In addition, worry, anhedonia, COVID-19-related fears, and contamination fears could be also alleviated [94]. Thus, internet-based interventions may offer a viable and scalable means of mitigating the rising mental health problems during the pandemic.

### Physiotherapy

In addition to pharmacological and psychological treatment, physiotherapy is an alternative or complementary therapy [79]. Prior evidence has confirmed the efficacy of neuromodulation on depression, and the positive effect and minimal adverse effects make it acceptable for patients with mental health problems [79, 95]. In addition, as part of a healthy lifestyle, exercise has been reported to be a fundamental intervention for depression and anxiety.

In one study, researchers applied two radio electric asymmetric conveyer (REAC) technology neuromodulation treatments to reduce psychosocial unease. They found that the REAC treatment helped patients with COVID-19 utilize better coping strategies to deal with environmental stress and relieve depressive and anxiety symptoms [96]. Shinjo *et al*. reported that bifrontal transcranial direct current stimulation (tDCS) significantly reduced the severe anxiety of a patient with COVID-19 [97]. Although no random-blind controlled trials conducted during the pandemic have been reported, expert consensus considers tDCS to be a potential treatment for mental distress associated with the COVID-19 epidemic [97, 98].

A large body of evidence has suggested that regular exercise significantly reduces the risk of depression and anxiety, and it is considered to be beneficial in the prevention of about 25 conditions [99–102]. To avoid spreading the virus, a balance of outdoor and indoor physical exercise should be maintained. Nagarathna *et al*. reported that yoga practice is a beneficial indoor exercise that reduced stress and anxiety and enhances immunity [103].

Based on these reports, different interventions can be selected according to different periods of the pandemic. For example, tDCS or REAC can be used to treat hospitalized COVID-19 patients. For close contacts living together, yoga practice or other indoor exercise can be performed during the period of isolation. Compared to exercising indoors, exercising outdoors brings about greater feelings of revitalization and positive engagement, as well as a decrease in depression and anxiety [104]. Therefore, for the general population, outdoor exercise is recommended.

## Conclusions

In this review, we described several factors contributing to depression and anxiety during the COVID-19 pandemic. For those populations at high risk of developing mental problems, psychological examinations should be conducted and more family and social support provided. Pathogenesis studies focused on neuroimaging, neuroimmune, and intestinal microbiology have identified potential biomarkers for the identification and diagnosis of mental disorders during the pandemic. Furthermore, new intervention targets for specific aberrant brain functions and immunity are promising. Drugs, psychological therapy, and physiotherapy are the three most commonly-used options for the treatment of mental disorders. However, due to the particulars of quarantine policies, treatment plans must be adjusted and optimized to a given situation. For patients with severe depression and anxiety, antidepressants ad antianxiety drugs should be prescribed. The options are more diverse for patients with moderate or mild mental health problems, such as tDCS or REAC for hospitalized COVID-19 patients, yoga or other indoor exercise for quarantined people, and outdoor exercise for the general populations. Overall, this review provides a reference for the identification, diagnosis, and optimized treatment of patients with depression and anxiety, with the goal of minimizing the adverse impact of COVID-19 on human well-being.
